# Local administration of low-intensity vibration improves wound healing in diabetic mice

**DOI:** 10.3389/fbioe.2026.1731310

**Published:** 2026-02-06

**Authors:** Rita E. Roberts, Jacqueline Cavalcante-Silva, Onur Bilgen, Rhonda D. Kineman, Timothy J. Koh

**Affiliations:** 1 Department of Kinesiology and Nutrition, University of Illinois at Chicago, Chicago, IL, United States; 2 Center for Tissue Repair and Regeneration, University of Illinois at Chicago, Chicago, IL, United States; 3 Jesse Brown VA Medical Center, Chicago, IL, United States; 4 Department of Mechanical & Aerospace Engineering, Rutgers University, Piscataway, NJ, United States; 5 Department of Medicine, Section of Endocrinology, Diabetes and Metabolism, University of Illinois at Chicago, Chicago, IL, United States

**Keywords:** angiogenesis, diabetes, growth factors, skin wound healing, vibration therapy

## Abstract

**Introduction:**

Chronic wounds related to diabetes incur significant morbidity and mortality, yet few effective therapies are available. Although whole body low-intensity vibration (LIV) has been shown to improve angiogenesis and wound healing in diabetic mice, local application of LIV signals could enhance the utility of this therapeutic approach.

**Methods:**

The purpose of the present study was to compare the effectiveness of different treatment regimens involving local LIV applied to wounds of diabetic mice via oscillating motor, or by a wearable piezoelectric device.

**Results:**

Local LIV delivered by oscillating motor enhanced angiogenesis, granulation tissue formation and wound closure to a similar degree for all vibration protocols tested. In addition, local LIV induced protocol-dependent increases in wound IGF1 and VEGF levels that did not necessarily correlate with the effects on healing. LIV delivered by piezoelectric disks involved accelerations that were an order of magnitude smaller than those delivered by the oscillating motor, but produced significant increases in angiogenesis and granulation tissue formation, with trends of enhanced wound closure. These changes were associated with increased VEGF but not IGF1 levels.

**Discussion:**

These findings demonstrate that local delivery of LIV can enhance key aspects of diabetic wound healing, highlighting its potential as a non-invasive method for improving healing of chronic diabetic wounds.

## Introduction

Chronic wounds associated with diabetes are an escalating problem worldwide. More than 8% of the adult population worldwide suffers from diabetes and associated complications, including chronic wounds (Chatterjee et al., 2017). In the United States, Medicare costs associated with chronic wounds have approached $13 billion per year (Geraghty and LaPorta, 2019; Magliano et al., 2019). People with diabetes incur a 25% lifetime risk of developing chronic wounds, which frequently lead to amputation, decreased quality of life, and high morbidity and mortality (Ramsey et al., 1999; Jeffcoate and Harding, 2003; Hoffstad et al., 2015). Wound healing requires coordinated responses of different cell types during the overlapping phases of inflammation, proliferation, and remodeling (Koh and DiPietro, 2011; Eming et al., 2014), and chronic wounds are known to exhibit defects in each phase of healing (Falanga, 2005; Blakytny and Jude, 2006). However, few therapies are available to target multiple phases of healing and improve the healing of diabetic wounds.

Energy-based treatment modalities, including laser, electrical, or mechanical stimulations, are often used in conjunction with standard treatments to improve angiogenesis and healing of chronic diabetic wounds (Glass et al., 2014; Ennis et al., 2016). Recently, we demonstrated that whole-body low-intensity vibration (LIV) can reduce markers of inflammation, improve angiogenesis, and accelerate wound closure in diabetic mice, in part by increasing growth factors such as insulin-like growth factor (IGF)-1 and vascular endothelial growth factor (VEGF) in the wound (Weinheimer-Haus et al., 2014; Roberts et al., 2021a; Roberts et al., 2023). In addition, it has been demonstrated that LIV increases skin blood flow (Nakagami et al., 2007; Maloney-Hinds et al., 2008; Tzen et al., 2018; Zhu et al., 2020) and inhibits the progression of pressure ulcers (Arashi et al., 2010; Sari et al., 2015). However, the mechanisms by which LIV signals promote different aspects of wound healing remain to be elucidated.

Local application of LIV signals represents an appealing method to improve the healing of diabetic wounds as, if effective, this approach would likely engender higher patient adherence at a lower cost than whole-body LIV. A recent study provided proof of concept for this idea, reporting that LIV delivered at ∼50 Hz with ∼0.1 mm displacement, either using an oscillating motor in anesthetized animals (Haba et al., 2023b) or via a bandage containing four mini-vibration motors, accelerated wound healing in awake animals (Haba et al., 2023a) using a rat model of hyperglycemia (streptozotocin-treated rats). In this study, we compared the effectiveness of treatment regimens involving local LIV applied using an oscillating motor every day versus every other day and regimens involving two different intensities of LIV on wound healing in a mouse model of type 2 diabetes (db/db mice). We also evaluated the effectiveness of LIV signals delivered using a wearable piezoelectric device on wound healing in these mice. Our findings demonstrate that local delivery of LIV can enhance key aspects of diabetic wound healing, highlighting its potential as a non-invasive method for improving the healing of diabetic wounds.

## Methods

### Animals

Diabetic db/db mice (BKS.Cg-Dock7m +/+ Leprdb/J) were obtained from the Jackson Laboratory. Experiments were performed on 12–16 week-old male mice. Only mice with fasting blood glucose >250 mg/dl were included in the study. Mice were housed under environmentally controlled conditions, with a 12-h light/dark cycle. Water and food were available *ad libitum*. To minimize bias, mice were randomly assigned to experimental groups, and the resulting samples were coded and analyzed in a blinded fashion. For all experimental procedures, mice were anesthetized with isoflurane (3% for induction and 2% for maintenance). For euthanasia, mice were anesthetized with 3% isoflurane, followed by cervical dislocation. All procedures involving animals were approved by the Animal Care Committee at the Jesse Brown Veterans’ Affairs Medical Center.

### Excisional wounding

Mice were subjected to excisional wounding, as described previously (Weinheimer-Haus et al., 2014; Roberts et al., 2021a; Roberts et al., 2023). In brief, mice were anesthetized using isoflurane, and their dorsum was shaved and cleaned with alcohol. Four 8-mm wounds, spaced ∼5 mm apart, were made on the back of each mouse using a dermal biopsy punch and covered with Tegaderm (3M, Minneapolis, MN) to keep the wounds moist and maintain consistency with the treatment of human wounds. Wound closure was assessed for all four wounds from each of three mice (N = 12 total). Two wounds from each of three mice were analyzed for each histological and growth factor assay (N = 6 total for each assay).

### Local LIV

**FIGURE 1:**
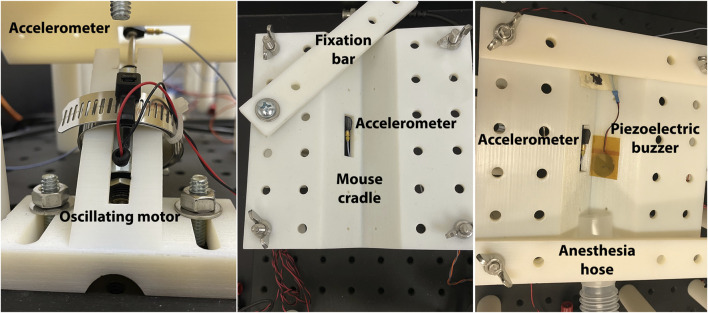
Experimental set-up for local delivery of LIV by oscillating motors or piezoelectric disks. Mice were anesthetized, placed on their back in a cradle, and fixed in place with a fixation bar. LIV was delivered through an oscillating motor or piezoelectric disk controlled using custom software and accelerations measured using an accelerometer.

All mice receiving local LIV treatment were anesthetized using isoflurane and then placed on their backs on the experimental platform (Figure 1). For the first set of experiments, local LIV was applied near the wounds via an indenter driven by an oscillating motor. Mice were randomly assigned to groups receiving one of two LIV protocols based on our previous studies on whole-body LIV (Weinheimer-Haus et al., 2014; Roberts et al., 2021a); the first involved application of LIV with low frequency (45 Hz) and acceleration (0.3 g) (LL), and the second involved high frequency (90 Hz) and acceleration (1.0 g) (HH). Accelerations were measured using an accelerometer placed on the tip of the indenter, and inputs to the oscillating motor were controlled using custom software. Each group was further split into two subgroups, with one subgroup receiving LIV daily and the second subgroup receiving LIV every other day. For mice that received LIV daily, treatment started on the day of injury (day 0) and ended on day 9. For mice that received LIV every other day, treatment occurred on the day of injury (day 0), day 2, day 4, day 6, and day 8. Mice were euthanized, and wounds were harvested on day 10. Control mice were anesthetized and placed in the experimental setup but did not receive LIV treatment.

**FIGURE 2:**
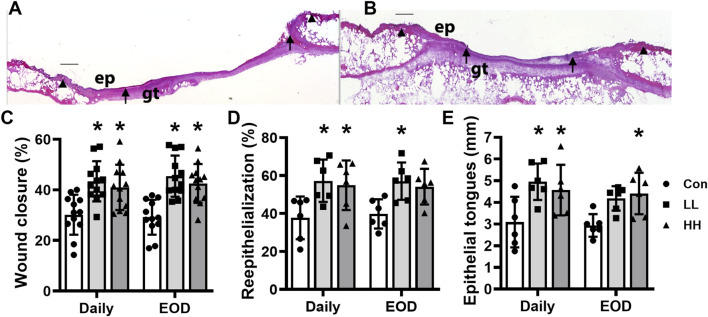
Local LIV applied by an oscillating motor promotes wound closure. Mice were treated with one of two LIV protocols: low frequency (45 Hz) and acceleration (0.3 g) (LL) or high frequency (90 Hz) and acceleration (1.0 g) (HH). Each group was divided into two subgroups; one received LIV treatment daily (Daily) and the second received LIV treatment every other day (EOD). Control (Con) mice were anesthetized but did not receive LIV treatment. **(A,B)** Representative images of hematoxylin and eosin-stained sections for **(A)** sham control and **(B)** LIV-treated wounds on day 10 post-wounding; arrows mark ends of epithelial tongues growing into wound, and arrowheads mark wound edges; ep, epithelium; gt, granulation tissue; scale bar = 500 μm. **(C)** Wound closure assessed in digital images of the surface of day 10 wounds. **(D)** Re-epithelialization measured in hematoxylin and eosin-stained sections from the center of day 10 wounds. **(E)** Length of epithelial tongues, summed across the two sides of the wound, measured in hematoxylin and eosin-stained sections from the center of day 10 wounds. For wound closure measurements, four wounds from each of three mice were analyzed (N = 12). For histological measurements, two wounds from each of three mice were analyzed (N = 6). *Mean value significantly different from Con for that treatment group, *p* < 0.05.

For the next set of experiments, local LIV was delivered near the wounds via piezoelectric disks (Figure 2). After induction of anesthesia, piezoelectric disks were placed near the wounds, and input parameters were adjusted to deliver vibrations with a frequency of 90 Hz and an acceleration of 0.02 g; these were the largest accelerations that could be generated using these disks, likely due to the small mass and vibration characteristics of the disks. Accelerations were measured near the wound site using an accelerometer. Experimental mice received LIV daily, starting from the day of injury (day 0) and ending on day 9. Mice were euthanized, and wounds were harvested on day 10. Control mice were anesthetized and placed in the experimental setup but did not receive LIV treatment.

### Wound closure

Wound closure was assessed in digital images of the external wound surface taken immediately after injury and on days 2, 6, and 10 post-injury. The wound area was measured using Fiji Image J and expressed as a percentage of the area immediately after injury.

### Wound histology

Re-epithelialization and granulation tissue thickness were measured in cryo-sections taken from the center of the wound (found by serial sectioning through the entire wound) and stained with hematoxylin and eosin (Weinheimer-Haus et al., 2014; Roberts et al., 2021a; Roberts et al., 2023). Digital images were obtained using a Keyence BZ-X710 All-in-One Fluorescence Microscope (Keyence, Itasca, IL, United States), with a ×2 or ×20 objective, and analyzed using ImageJ software. The percentage of re-epithelialization, length of epithelial tongues, and granulation tissue area were measured in three sections per wound and averaged across sections to provide a representative value for each wound.

Angiogenesis was assessed by immunohistochemical staining for CD31 (390, 1:100; BioLegend, San Diego, CA, United States), macrophage accumulation by staining for F4/80 (BM8, 1:100, Thermo Fisher, Waltham, MA, United States), and neutrophil accumulation by staining for Ly6G (1A8, 1:100, BD Pharmingen, Franklin Lakes, NJ, United States). Collagen deposition was assessed using Masson trichrome staining (IMEB, San Marcos, CA, United States). For each assay, digital images were obtained covering the wound bed (2–3 fields using a ×20 objective), and the stained percentage area was quantified by the number of clearly stained pixels above a threshold intensity and normalized to the total number of pixels. The software application allowed the observer to exclude artifacts. For each assay, three sections per wound were analyzed.

### ELISA

Wounds that had been snap-frozen in liquid nitrogen and stored at −80 C were homogenized in cold PBS (10 μl of PBS per mg of wound tissue) supplemented with a protease inhibitor cocktail (Sigma Aldrich, St. Louis, MO, United States) using a Dounce homogenizer and then centrifuged. Supernatants were used for enzyme-linked immunoassays of IGF1 and VEGF (R&D Systems, Minneapolis, MN, United States).

### Statistics

Values are presented as the means ± standard deviation. For oscillating motor results, measurements of wound healing or wound growth factors were compared between low- and high-intensity LIV, daily and every-other-day, and sham control treatment groups using two-way ANOVA. Sidak’s multiple-comparisons test was used when ANOVAs demonstrated significance. For piezoelectric disk results, measurements of wound healing or wound growth factors were compared between LIV and sham treatment groups using t-tests. Differences between groups were considered significant if *p* ≤ 0.05.

## Results

### Local LIV applied using an oscillating motor promotes wound closure

Diabetic db/db mice were treated with one of four LIV protocols: low frequency with low acceleration (LL) or high frequency with high acceleration (HH), applied either daily or every other day. Over the course of the experiment, mice lost ∼5% body weight, which was similar across protocols, and none of the protocols altered blood glucose levels (Table 1). All the LIV protocols accelerated wound closure on day 10 post-injury, as assessed by external measurements, compared to sham controls (Figure 2). Similarly, all the LIV protocols increased histological measurements of re-epithelialization on day 10 post-injury compared to the sham control (Figure 2). There were little, if any, differences in the effects of the different LIV protocols on these measurements of epithelial closure. In short, locally applied LIV enhances wound closure in diabetic mice, independent of vibration intensity within the range tested in these experiments and of whether LIV was applied daily or every other day.

**TABLE 1:** Body weights and blood glucose levels for db/db mice in the oscillating motor LIV experiment.

Con/LIV	Timing	Pre-injury body weight (g)	Post-injury body weight (g)	Pre-injury blood glucose (mg/dl)	Post-injury blood glucose (mg/dl)
Con	ED	52.6 (1.5)	49.3 (1.3)	482.7 (65.2)	512.0 (73.6)
Con	EOD	51.3 (0.8)	48.5 (1.3)	500.0 (47.5)	494.3 (54.9)
LL	ED	52.8 (2.3)	50.5 (1.5)	512.7 (60.5)	487.7 (60.6)
LL	EOD	51.9 (0.9)	49.5 (1.1)	506.7 (57.5)	498.0 (71.3)
HH	ED	51.3 (1.4)	49.0 (1.5)	485.3 (49.0)	504.7 (50.1)
HH	EOD	50.6 (0.8)	48.7 (1.0)	495.3 (52.2)	506.3 (45.9)

### Local LIV applied using an oscillating motor promotes wound angiogenesis and granulation tissue formation

**FIGURE 3:**
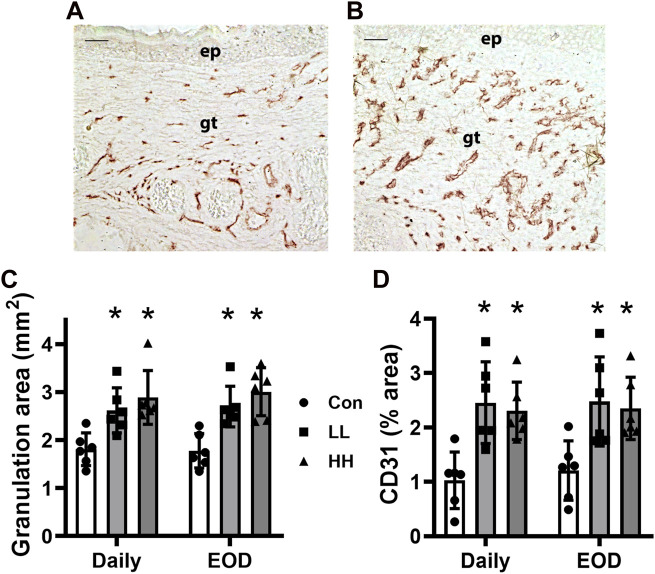
Local LIV applied by an oscillating motor promotes wound angiogenesis and granulation tissue formation. Mice were treated with one of two LIV protocols: low frequency (45 Hz) and acceleration (0.3 g) (LL) or high frequency (90 Hz) and acceleration (1.0 g) (HH). Each group was divided into two subgroups; one received LIV treatment daily (daily), and the second received LIV treatment every other day (EOD). Control (Con) mice were anesthetized but did not receive LIV treatment. **(A,B)** Representative images of CD31-stained sections for **(A)** sham control and **(B)** LIV-treated wounds on day 10 post-wounding; ep, epithelium; gt, granulation tissue; scale bar = 50 μm. **(C)** Granulation tissue thickness measured in hematoxylin and eosin-stained sections from center of day 10 wounds. **(D)** Angiogenesis was measured as the percent area stained with the antibody against CD31 in sections from the center of day 10 wounds. Two wounds from each of three mice were analyzed for each histological assay (N = 6 total). *Mean value significantly different from Con for that treatment group, *p* < 0.05.

Similarly, all the LIV protocols increased the granulation tissue area (Figure 3, example images in Figure 2) and enhanced angiogenesis assessed by CD31 staining (Figure 3), on day 10 post-injury compared to sham controls. Again, there were little, if any, differences in the effects of the different LIV protocols on these measurements of dermal healing. In short, locally applied LIV enhances angiogenesis and granulation tissue formation in wounds of diabetic mice, independent of vibration intensity within the range tested and of whether LIV was applied daily or every other day.

### Local LIV applied using an oscillating motor increases wound growth factor levels

**FIGURE 4:**
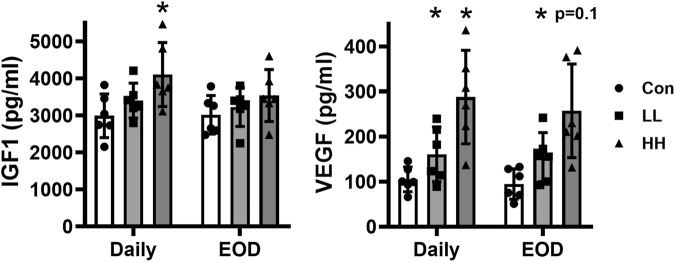
Local LIV applied by an oscillating motor increases wound growth factor levels. Mice were treated with one of two LIV protocols: low frequency (45 Hz) and acceleration (0.3 g) (LL) or high frequency (90 Hz) and acceleration (1.0 g) (HH). Each group was divided into two subgroups; one received LIV treatment daily (daily), and the second received LIV treatment every other day (EOD). Control (Con) mice were anesthetized but did not receive LIV treatment. Protein levels of IGF-1 and VEGF measured in homogenates of day 10 wounds using ELISA. Two wounds from each of three mice were analyzed for each assay (N = 6 total). *Mean value significantly different from Con of that treatment group, *p* < 0.05.

Our previous studies on whole-body LIV showed that LIV-enhanced wound healing was associated with increased wound IGF1 levels in obese and diabetic db/db mice (Weinheimer-Haus et al., 2014; Roberts et al., 2021a). Importantly, knockdown of liver IGF1 blunted the whole-body LIV-induced increase in wound IGF1 levels and improved healing in wounds of high-fat diet-fed obese and insulin-resistant mice (Roberts et al., 2023). In contrast to these studies, locally applied LIV only increased wound IGF1 levels when applied daily at high intensity; the other LIV protocols did not increase IGF1 levels, despite enhancing wound healing (Figure 4). In addition, local LIV induced an intensity-dependent increase in wound VEGF levels whether applied daily or every other day (Figure 4). Therefore, although specific protocols of locally applied LIV increased IGF1 and VEGF levels, other factors likely contribute to local LIV-enhanced wound healing, as shown in Figures 2, 3.

### Local LIV delivered by piezoelectric disks enhances wound closure

**FIGURE 5:**
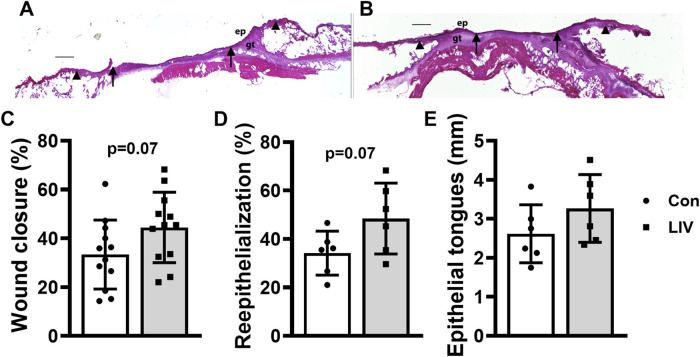
Local LIV applied by a piezoelectric disk may enhance wound closure. Mice were treated daily with local LIV delivered by a piezoelectric disk. Control (Con) mice were anesthetized but did not receive LIV treatment. **(A,B)** Representative images of hematoxylin and eosin-stained sections for **(A)** sham control and **(B)** LIV-treated wounds on day 10 post-wounding; arrows mark ends of epithelial tongues growing into wound, and arrowheads mark wound edges; ep, epithelium; gt, granulation tissue; scale bar = 500 μm. **(C)** Wound closure assessed in digital images of the surface of day 10 wounds. **(D)** Re-epithelialization measured in hematoxylin and eosin stained-sections from the center of day 10 wounds. **(E)** Length of epithelial tongues, summed across the two sides of the wound, measured in hematoxylin and eosin-stained sections from the center of day 10 wounds. For wound closure measurements, four wounds from each of three mice were analyzed (N = 12 total). For histological measurements, two wounds from each of three mice were analyzed (N = 6 total). *Mean value significantly different from Con for that treatment group, *p* < 0.05.

To advance our studies toward a wearable device, diabetic mice were treated daily with LIV delivered by piezoelectric disks at a frequency of 90 Hz and an acceleration of 0.02 g. This was the largest acceleration achievable with these disks. As with the oscillating motor experiments, mice lost <5% body weight, and blood glucose levels were not altered over the course of the experiment (Table 2). LIV delivered by piezoelectric disks showed a trend of accelerating wound closure on day 10 post-injury, as assessed by external measurements, compared to sham controls (Figure 5). Similarly, LIV delivered by piezoelectric disks showed a trend of increasing histological measurements of re-epithelialization compared to sham controls (Figure 5). In short, LIV delivered by piezoelectric disks shows potential for enhancing wound closure, although the small accelerations delivered by this method may have limited the efficacy of treatment.

**TABLE 2:** Body weights and blood glucose levels for db/db mice in the piezoelectric disk LIV experiment.

Con/LIV	Timing	Pre-injury body weight (g)	Post-injury body weight (g)	Pre-injury blood glucose (mg/dl)	Post-injury blood glucose (mg/dl)
Con	ED	49.1 (1.4)	47.1 (1.6)	523.0 (63.0)	518.3 (40.0)
LIV	ED	49.5 (2.5)	47.4 (1.7)	505.2 (55.9)	517.2 (52.1)

### Local LIV delivered by piezoelectric disks promotes wound angiogenesis and granulation tissue formation

**FIGURE 6:**
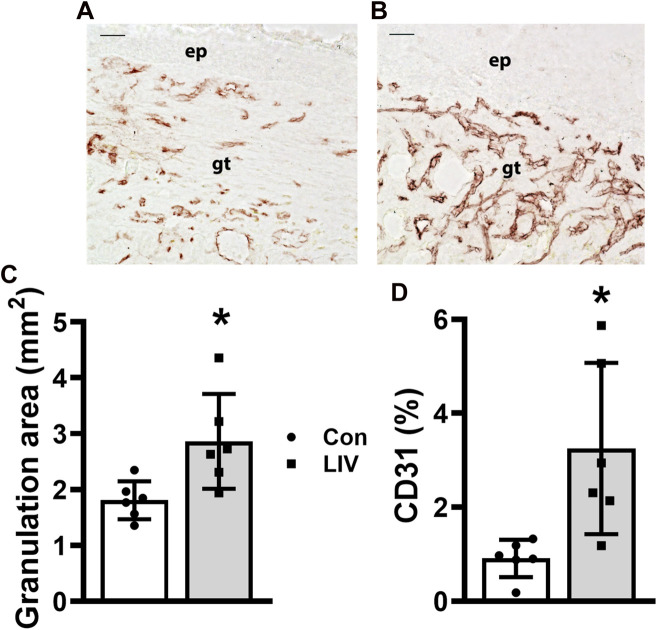
Local LIV applied by a piezoelectric disk promotes wound angiogenesis and granulation tissue formation. Mice were treated daily with local LIV delivered by a piezoelectric disk. Control (Con) mice were anesthetized but did not receive LIV treatment. **(A,B)** Representative images of CD31-stained sections for **(A)** sham control and **(B)** LIV-treated wounds on day 10 post-wounding; ep, epithelium; gt, granulation tissue; scale bar = 50 μm. **(C)** Granulation tissue thickness measured in hematoxylin and eosin-stained sections from the center of day 10 wounds. **(D)** Angiogenesis was measured as percent area stained with the antibody against CD31 in sections from the center of day 10 wounds. Two wounds from each of three mice were analyzed for each assay (N = 6 total). *Mean value significantly different from Con for that treatment group, *p* < 0.05.

Importantly, LIV delivered by piezoelectric disks significantly increased the granulation tissue area on day 10 post-injury (Figure 6, example images in Figure 5) and increased angiogenesis assessed by CD31 staining (Figure 6) compared to sham controls. Therefore, local LIV delivered by piezoelectric disks appears to have a stronger impact on dermal healing than epidermal healing of wounds in diabetic mice.

### Local LIV delivered by piezoelectric disks increases wound growth factor levels

**FIGURE 7:**
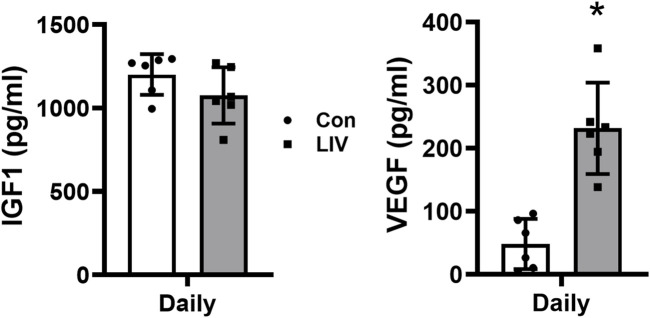
Local LIV applied by a piezoelectric disk increases wound VEGF levels. Mice were treated daily with local LIV delivered by a piezoelectric disk. Control (Con) mice were anesthetized but did not receive LIV treatment. Protein levels of IGF-1 and VEGF measured in homogenates of day 10 wounds using ELISA. Two wounds from each of three mice were analyzed for each assay (N = 6 total). *Mean value significantly different from Con of that treatment group, *p* < 0.05.

In contrast to our previous studies on whole-body LIV (Weinheimer-Haus et al., 2014; Roberts et al., 2021a) and our experiments with local LIV applied using an oscillating motor (Figure 4), locally applied LIV delivered by piezoelectric disks did not increase wound IGF1 levels (Figure 7). In contrast, LIV delivered by piezoelectric disks markedly increased wound VEGF levels (Figure 7). Therefore, the production of VEGF in wounds appears to be more sensitive to local LIV signals than IGF1.

## Discussion

We aimed to compare the effectiveness of different local LIV treatment regimens on wound healing in diabetic mice. Local LIV delivered by an oscillating motor enhanced angiogenesis, granulation tissue formation, and wound closure, independent of the vibration intensities tested or application frequencies tested (daily or every other day). In addition, local LIV induced protocol-dependent increases in wound IGF1 and VEGF levels did not consistently correlate with effects on healing. LIV delivered by piezoelectric disks involved accelerations that were more than 10 times smaller than those delivered by the oscillating motor, but it produced significant increases in angiogenesis and granulation tissue formation, with trends toward enhanced wound closure. These effects were associated with increased VEGF levels, but not IGF1 levels. Differences in device–tissue interactions, including contact mechanics and local strain distribution, likely contributed to the observed differences between the effects of LIV delivered by the oscillating motor versus those by piezoelectric disks. In summary, our findings demonstrate that local delivery of LIV can enhance key processes in diabetic wound healing, highlighting its potential as a non-invasive method for improving the healing of chronic diabetic wounds.

Energy-based wound therapies, including ultrasound, laser, electrical, and mechanical stimulations, can be used to improve angiogenesis and the healing of diabetic wounds (Glass et al., 2014; Ennis et al., 2016). Local application of LIV signals represents an appealing approach as this could be incorporated into a low-cost wearable bandage (Shultis et al., 2021; Haba et al., 2023a) that would likely increase patient adherence compared to other approaches. Recent studies provided proof of concept for this idea, reporting that LIV delivered daily for 40 min at ∼50 Hz either via an oscillating motor in anesthetized animals (Haba et al., 2023b) or via a bandage containing four mini-vibration motors accelerated wound healing in awake animals (Haba et al., 2023a) using a rat model of hyperglycemia (streptozotocin-treated rats). Both modes of LIV increased granulation tissue formation and accelerated wound closure, associated with increased VEGFA protein levels. Interestingly, in this prior study, for LIV delivered by an oscillating motor, lower-intensity LIV was superior to higher-intensity LIV in promoting wound healing. This contrasts with our results, showing little difference in wound healing outcomes for the LIV accelerations tested. Unfortunately, comparison of LIV intensities between studies is difficult as the previous study reported intensities as the peak-to-peak mV driving the oscillating motor, whereas we measured the accelerations produced by the indenter of our motor.

Regarding potential mechanisms underlying LIV-enhanced wound healing, a common finding of our current and previous studies is that a variety of LIV protocols, delivered either to the whole body or locally to the wound, increased VEGF levels, which were associated with enhanced angiogenesis in the wound (Weinheimer-Haus et al., 2014; Haba et al., 2023a; Haba et al., 2023b). In addition, it has been demonstrated that both local and whole-body application of LIV acutely increases skin blood flow (Nakagami et al., 2007; Maloney-Hinds et al., 2008; Tzen et al., 2018; Zhu et al., 2020), which could represent another common mechanism contributing to improved healing. We also found that whole-body LIV increases wound IGF1 levels in diabetic db/db mice (Weinheimer-Haus et al., 2014; Roberts et al., 2021a), and we recently demonstrated that whole-body LIV also increases IGF1 levels in the liver and blood; since the liver is a major source of IGF1 in wounds (Roberts et al., 2021b), we focused on the role of liver IGF1 in LIV-enhanced healing. We found that knockdown of IGF1 in the liver blunts LIV-induced improvements in wound healing in pre-diabetic and obese HFD mice (Roberts et al., 2023). However, IGF1 levels were not consistently increased by our local LIV protocols in wounds, or blood, or liver (data not shown), suggesting that the mechanisms underlying improved healing may differ between modes of LIV application.

A limitation of our study is that we have not yet optimized the therapeutic potential of the piezoelectric disk approach. Although the small accelerations generated by the current small disks were able to improve angiogenesis and granulation tissue formation, we envision testing larger disks or arrays of smaller disks that should produce larger accelerations for human studies. Evaluating these protocols over a broader range of time points would also provide additional insights into the impact on healing kinetics, particularly for late-stage healing and remodeling. In addition, our assessment of angiogenesis was limited to the percentage area stained with the endothelial cell marker CD31. In-depth analysis of new vessel localization, lumen formation, pericyte coverage, and vessel functionality would be an excellent focus for future research. Moreover, although LIV-enhanced wound healing is associated with increased wound VEGF levels, neither we nor others have demonstrated a mechanistic link between the two. Furthermore, it is unlikely that only one pathway contributes to LIV-enhanced healing. Therefore, we have an ongoing project to characterize the gene expression changes induced by LIV at the single-cell level, along with potential interactions between inflammatory cells and other wound cells, to better understand the holistic wound response to LIV signals. Finally, it remains unclear how LIV, whether applied to the whole body or locally to the wound, compares to other energy-based treatment modalities, including laser, electrical, or other forms of mechanical stimulation, in terms of effects on wound healing or mechanisms involved.

In conclusion, local delivery of LIV can enhance key aspects of diabetic wound healing, including enhanced angiogenesis, granulation tissue formation, and wound closure, highlighting its potential as a non-invasive method for improving the healing of chronic diabetic wounds.

## Data Availability

The datasets presented in this study can be found in online repositories. The names of the repository/repositories and accession number(s) can be found at https://figshare.com/s/389169e053c73349b118.
